# Inflammation and Tumor Microenvironment in Lymph Node Metastasis

**DOI:** 10.3390/cancers3010927

**Published:** 2011-03-01

**Authors:** Xuesong Wu, Tomonori Takekoshi, Ashley Sullivan, Sam T. Hwang

**Affiliations:** Department of Dermatology, Medical College of Wisconsin, Milwaukee, WI 53226, USA; E-Mails: xwu@mcw.edu (X.W.); ttakekoshi@mcw.edu (T.T.); ansullivan@gmail.com (A.S.)

**Keywords:** melanoma, lymph node metastasis, microenvironment, inflammation, cytokines, chemokines, epithelial-mesenchymal transition, lymphangiogenesis

## Abstract

In nearly all human cancers, the presence of lymph node (LN) metastasis increases clinical staging and portends worse prognosis (compared to patients without LN metastasis). Herein, principally reviewing experimental and clinical data related to malignant melanoma, we discuss diverse factors that are mechanistically involved in LN metastasis. We highlight recent data that link tumor microenvironment, including inflammation (at the cellular and cytokine levels) and tumor-induced lymphangiogenesis, with nodal metastasis. Many of the newly identified genes that appear to influence LN metastasis facilitate general motility, chemotactic, or invasive properties that also increase the ability of cancer cells to disseminate and survive at distant organ sites. These new biomarkers will help predict clinical outcome and point to novel future therapies in metastatic melanoma as well as other cancers.

## 1. Introduction

Melanoma is a clinically heterogeneous cancer derived from melanocytes, the pigment-producing cells of the skin and eye [1]. Although the majority of patients have thin local lesions that are surgically resectable with a very high probability of cure, certain patients have lesions that disseminate either to regional lymph nodes or to visceral organs, resulting in aggressive metastatic disease. Patients presenting with organ metastases have five-year survival rates of less than 10% [2]. There are few treatment options for melanoma once it has metastasized since the disease is refractory to existing chemotherapeutic drugs and only partially responsive to immunotherapy.

Lymph node (LN) metastasis in melanoma plays a strong predictive role in assessing patient survival. In the recently updated 2010 TNM staging system, nodal tumor deposits of any size are included in staging, affirming the importance of regional nodal metastasis in patient prognosis [3]. While it is clear that LN metastasis in melanoma is predictive of poor patient outcome, it is not clear if LN metastasis is merely a marker for melanoma cells that have high metastatic capacity or whether LN metastasis itself has a causal role and precedes metastatic melanoma to visceral sites. It is known that patients with positive nonsentinel LN have a higher risk of distant metastases [4], but those with isolated metastatic cells (<0.01 mm) in sentinel LN do not have a worse prognosis than patients with no detectable sentinel LN metastasis, suggesting that small numbers of sentinel LN micrometastases do not portend distant metastases [5]. The well-characterized sequential nature of malignant melanoma progression and the availability of animal models make this disease an excellent system for studying the molecular changes associated with the metastatic phenotype. In this review, we discuss those important factors in early stage nodal metastasis and their prognostic significance in melanoma patients by highlighting recent data that link inflammation (at the cellular and cytokine levels), the tumor microenvironment (including epithelial-to-mesenchymal transition), and nodal metastasis. We also discuss the role of a particularly important family of trafficking receptors, namely the chemokine receptors, as well as the roles of tumor-induced lymphangiogenesis in LN metastasis. These studies identify new biomarkers that may help predict clinical outcome and point to novel future therapies in metastatic melanoma.

## 2. Clinical Implications of Nodal Metastasis in Melanoma

Analysis of sentinel lymph nodes (SLN) is a major determinant for the staging and clinical management of melanoma. Approximately 50% of all cutaneous melanoma patients with tumor progression first develop regional LN metastases. In the other 50%, the initial metastases are either satellite/in-transit metastases (∼20%) or immediate distant metastases (∼30%). Furthermore, there is a certain percentage of transition from satellite/intransit metastasis to both regional LN and distant metastasis and from regional LN to distant metastasis [6].

Although different opinions exist as to its impact on patient outcome, selective sentinel lymph node dissection (SLND) is widely performed on cutaneous melanoma patients without clinical evidence of nodal metastases (*i.e.*, no palpable enlargement of SLN draining the tumor site). The prognostic utility of SLN dissection is supported by a series of patients who underwent melanoma primary excision and SLN mapping. In this investigation, the presence of SLN metastasis correlated significantly with primary tumor thickness and ulceration [7]. Furthermore, the five-year disease free survival (DFS) and overall survival (OS) rates of SLN tumor-positive patients were significantly lower than those DFS and OS rates of SLN tumor-negative patients [7,8]. The SLN-positive patient overall survival rate was found to be superior to that OS rate reported for stage III patients who were treated with curative nodal dissection only after palpable adenopathies were appreciated on physical exam [8]. Other studies have also demonstrated that patients with SLND exhibit significantly lower rates of recurrence and distant metastases compared to those without surgical intervention [9,10]. Despite these data affirming the prognostic value of detection of LN metastases in melanoma, the overall survival advantage of elective LN dissection or sentinel LN biopsy in clinically node-negative individuals is unclear, in part, due to particular difficulties in interpreting clinical prognostic data in head and neck melanoma vs. those of other anatomic sites [11,12]. Specifically, the complex lymphatic drainage of the head and neck results in poor detection of draining LN in that region, resulting in a high false-negative identification rate of involved LN [12].

Metastasis beyond the SLN to the nonsentinel nodes (NSN) has been validated as a predictor of survival by several clinical studies. In one study, a total of 2,335 patients were analyzed for DFS and OS rates as a measure of favorable outcomes over a mean follow-up of 68 months. Three groups were compared: SLN negative (n = 1988), SLN-only positive (n = 296), and both SLN and NSN positive (n = 51). The five-year DFS rates were 85.5, 64.8, and 42.6% respectively (P < 0.001), and the five-year OS rates were 85.5, 64.9, and 49.4%, respectively (P < 0.001) [13]. By utilizing multivariate analysis, the presence of positive NSN in patients with existing positive SLN biopsies was shown to be statistically significantly associated with poorer outcomes independent of other risk factors, *i.e.*, increasing age, male sex, breslow depth, presence of extracapsular extension in SLN, and a positive NSN [14,15]. This data supports the concept that LN metastasis, whether to the initial draining LN or to NSN, is a sign of worsened patient survival.

## 3. Tumor Microenvironment Contributes to the Malignant Phenotype of Melanoma and May Positively or Negatively Regulate Metastasis

Recent studies have documented that only a small fraction of cancer cells can metastasize once they leave the primary tumor. When this occurs, malignant cells leave their primary site and disseminate by various routes, such as the blood and lymph vessels. Successful metastasis depends both on the intrinsic properties of the tumor cells and on the factors derived from the tumor microenvironment [16]. For instance, the microenvironment provides ample access to both blood and lymphatic vessels in and around the tumor, an inflammatory milieu consisting of immune cells and their secretory products, and a scaffold in the form of an extracellular matrix [17]. Although malignant melanoma had been traditionally conceptualized as a cell-autonomous event, increasing evidence supports the notion that these tumors are not isolated entities. Instead, they depend on, interact with, and react to the adjacent microenvironment [18].

Pathophysiological characteristics of tumor microenvironment include the lack of nutrients or oxygen, a low pH, and the production of pro-inflammatory mediators [19]. Clinical studies have shown that presence of hypoxia within a tumor is an independent marker of poor prognosis [20]. In one study, global transcriptional profiling of a panel of metastatic melanoma cell lines revealed that increased expression of genes activated in response to hypoxia, such as PLAUR (uPAR, the gene encoding the receptor for urokinase plasminogen activator, with roles in localizing and promoting plasmin formation, influencing cell-surface plasminogen activation and degrading extracellular matrix) and HIF1 (Hypoxia-inducible factor 1), was associated with cell lines with higher rates of metastasis [1]. The functional importance of uPAR was validated by the observation that hypoxia promoted spontaneous lymph node metastasis in human melanoma xenografts [21]. In addition, there was a strong histological correlation between uPAR-positive cells and areas of hypoxia in individual tumors. Metastatic tumors showed significantly higher hypoxia and uPAR-positive levels than nonmetastatic tumors of the same size. Moreover, inhibition of uPAR with monoclonal antibodies prevented metastasis almost completely [21].

Clinically, those patients with recurrent local disease following radiation therapy have an increased probability of developing regional and distant metastases [22]. The mechanisms underlying this phenomenon were studied by using R-18 human melanoma xenografts grown in preirradiated host sites in BALB/c-nu/nu mice. The metastatic frequency was higher in tumors in preirradiated beds than in control tumors in non-irradiated beds, and this prometastatic effect increased with preirradiation dosing. Strong positive correlations were found between the degree of hypoxia, presence of uPAR-positive cells, and metastatic frequency. Thus, primary tumors recurring after inadequate radiation therapy showed increased metastatic propensity due to hypoxia and hypoxia-induced up-regulation of metastasis-promoting gene products, including uPAR and HIF1a [23,24].

## 4. Increasing Evidence Supports Inflammation-Driven Involvement in Melanoma Lymph Node Metastasis

Inflammation in the tumor-microenvironment has gained prominent attention as a potential critical player in tumor metastasis. An inflammatory tumor microenvironment fosters tumor growth, angiogenesis, and metastasis progression. Cytokines play critical roles in promoting inflammation. In human melanoma cells, tumor necrosis factor-α (TNF-α) has been reported to upregulate integrin expression, cell attachment, and invasion of cells through fibronectin. Imaging studies focusing on migrating ‘fronts’ of cells revealed that melanoma cells responded with a significant increase in migration distance when stimulated with TNF-α during an *in vitro* scratch migration assay. Interestingly, the actions of TNF-α can be suppressed by the addition of an anti-inflammatory peptide, α-melanocyte-stimulating hormone (α-MSH) [25].

Cyclooxygenase 2 (COX-2), an inducible enzyme involved in regulating inflammatory process, is up-regulated in both primary and metastatic melanoma. COX-2 has been demonstrated to be expressed by both melanoma cells as well as infiltrating inflammatory cells, and its expression has been correlated with disease progression [26]. COX-2 was present at extremely high levels in the lymph node metastases compared to primary cutaneous melanoma, which suggests a potential role of COX-2 in promoting melanoma nodal metastasis [27]. Epidemiological studies have suggested that the risk of developing or dying from cancer is reduced in individuals who were on long term treatment with nonsteroidal anti-inflammatory drugs (NSAIDs), such as aspirin. NSAIDs act by inhibiting prostaglandin (PG)-endoperoxide synthase enzymes in the PG synthesis pathway. Ibuprofen significantly reduced TNF-α-stimulated migration of melanoma cells [28]. Another NSAID, sodium salicylate, both inhibited TNF-α-stimulated NF-kB activation and downregulated ICAM-1 expression in melanoma cells. This marked reduction of melanoma invasion and migration by sodium salicylate indicated that NSAIDs may be a potential therapeutic approach to oppose inflammation-induced melanoma invasion and metastasis *in vivo* [29]. NSAIDs, however, have their own unique side effect profile which may limit long term use.

The interaction of melanoma cells with fibroblasts may be a key initial event in triggering the release of inflammatory cytokines and chemokines by fibroblasts. When co-cultured with human melanoma cell lines, gene expression of fibroblasts changes dramatically. Key chemokines and cytokines, such as IL-1 beta, IL-8, IL-6 and CCL2/MCP1, were significantly upregulated in fibroblasts co-cultured with the invasive melanoma lines (BLM and MV3) compared to fibroblasts co-cultured with noninvasive (WM164) cells. Targeting inflammatory cytokines, such as IL-1β, by siRNA in the melanoma-stimulated fibroblasts resulted in reduced melanoma cell invasion [30].

Of note, regional LNs that had been invaded by metastatic cells showed a different expression profile of inflammatory cytokines and chemokines. Low-density, focused microarrays were used to identify and then hierarchically cluster chemokines in tumor-positive sentinel lymph nodes, compared to tumor-negative sentinel nodes and nonsentinel nodes in melanoma. Expression levels of interleukin-13 (IL-13), leptin, lymphotoxin β receptor (LTbR), and macrophage inflammatory protein 1β (MTP1β) were significantly higher and expression level of IL-11Rα was lower for tumor-positive nodes as compared with tumor-negative SN. Analysis of the expression of five genes, including IL-13, leptin, LTbR, MTP1β, and IL-11Rα, suggested a high concordance between gene-expression profiles and SN staging. These changes may provide clues to the early tumor lymph node interaction and subsequent metastasis, which may eventually be developed as a useful biomarker for predicting prognosis [31].

## 5. Leukocyte Infiltration Assists Malignant Behavior of Melanoma Cells through Inflammatory Mechanisms

The principal cellular mediators in the inflammatory tumor microenvironment are macrophages. Tumor-associated macrophages (TAMs) represent a paradigm for the pro-tumor activity of inflammatory cells and their mediators. In addition to promoting carcinogenesis, TAMs and their released factors (e.g., cytokines) have long been known to support all steps of invasion and metastasis. For instance, IL-1 and TNF-α are potent stimulators of metastasis. The level of TAM density was reported to be significantly higher in thick (>0.75 mm) *versus* thin (≤0.75 mm) melanomas, and was positively correlated with both melanoma invasiveness and metastasis to LN or other sites [32]. In addition to cytokines, macrophages are also a source of extracellular matrix (ECM) proteins, (e.g., macrophage-derived SPARC, osteonectin), which are beneficial to tumor cells in angiogenesis, proliferation, and migration. As opposed to classically activated macrophages (M1), which have tumoricidal activity and elicit tissue-destructive reactions, TAMs undergo alternative (M2) activation in response to IL-4 or IL-13 that is oriented toward tissue repair, immunosuppression, and tumor promotion. TAMs express migration stimulating factor (MSF), which stimulates tumor cell migration, thereby mediating invasion and metastasis [33]. Moreover, TAMs are one of the sources of vascular endothelial growth factor-C (VEGF-C), which has been linked to lymphatic dissemination of tumor cells through lymphangiogensis [34].

Neutrophils are also contained in the inflammatory infiltrate found in malignant melanoma. They are observed throughout primary melanoma tumors, but their numbers are increased in thicker, invasive tumors and in ulcerated areas [35]. Neutrophil-mediated tumor progression appears to be associated with angiogenesis and basement membrane invasion. Tumor-associated neutrophils release a variety of proteases (e.g., MMP-9, type IV collagenase and heparanase), which degrade and remodel the ECM, facilitating angiogenesis and increasing the metastatic propensity [36–38]. Interleukin-8 (IL-8) regulates neutrophil mobilization and activity. Upon co-culture with melanoma cells, the pro-angiogenic factors IL-8, IL-6, and IL-1β are up-regulated in neutrophils, synergistically promoting a microenvironment favorable for melanoma invasiveness [39].

## 6. Chemokines and Chemokine Receptors Play a Role in Melanoma Invasiveness and LN Metastasis

Chemokines are low molecular weight, secretory proteins that bind to seven transmembrane G protein-coupled receptors (GPCR) [40]. Chemokines and/or chemokine receptors are often strongly up-regulated with malignant cells, which include melanoma. Melanoma tumor cells have been reported to produce multiple chemokines including CXCL1-3, CXCL5-8, CXCL10, CCL2, and CCL5; as well as the chemokine receptors CXCR1, CXCR2, CXCR3, CXCR4, CXCR6, CXCR7, CCR1, CCR2, CCR5, CCR7, CCR9, and CCR10 [41].

Analogous to leukocyte migration which is guided by chemokine-chemokine receptor interactions, tumor cells utilize the chemokine signals to facilitate localization to proximal and even distant metastatic sites where cognate ligands are expressed. The mechanisms by which chemokine receptors on tumor cells facilitate LN metastasis are illustrated by studies on the roles of CCR7 and CCR10 in experimental metastasis of B16 murine melanoma cells [40]. CCR7 and CCR10 were two of only four chemokine receptors that were found to be upregulated in a series of melanoma cell lines [42], and CCR7 expression in melanoma cell lines and in tumors was further confirmed by Takeuchi *et al.* [43].

The role of CCR7 as a mediator of LN homing capacity in melanoma as well as other cancers was highly suspected based on the known physiologic role of CCR7 in the homing of dendritic cells to lymphatic vessels and to secondary lymphoid organs. The two known ligands for CCR7, CCL19 and CCL21, were both constitutively expressed by lymphatic endothelial cells and within the T cells zones of LN [44]. The functional role of CCR7 in tumor metastasis was studied by implanting B16 tumor cells that were transduced with CCR7 or empty vectors in mouse footpads [45]. Strikingly, ∼700-fold more mRNA for TRP (tyrosinase-related protein-1), a melanocyte-specific enzyme, was detected in draining LNs from CCR7-B16 cell-injected mice compared to LN from control animals. Furthermore, two weeks after implantation, 58% of the draining LNs from CCR7-B16 cells injected mice (*vs.* 5% of those from control mice) exhibited gross metastases. The LN metastasis of CCR7-B16 cells was blocked by neutralizing anti-CCL21, but not control, antibodies [45]. These data demonstrated that CCR7 expression was sufficient to substantially increase LN metastasis in melanoma cells that overexpressed this receptor. *In vivo* migration studies showed that CCR7 expression allows melanoma cells to migrate more efficiently toward depots of lymphatic endothelial cells [46,47].

**Figure 1.**
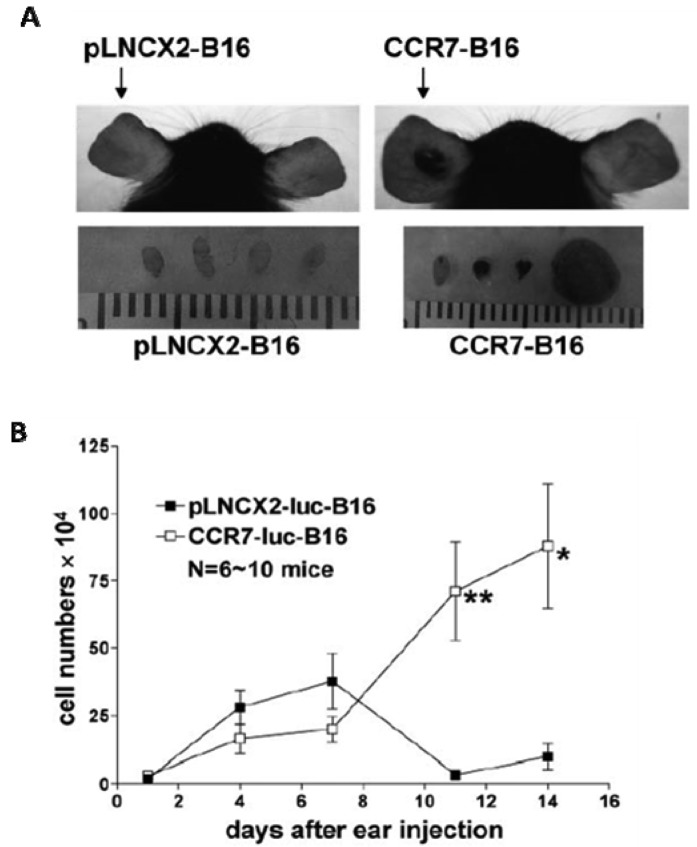
CCR7 facilitated B16 cell tumor formation and lymph node (LN) metastasis following ear skin inoculation. (**A**). Contrary to control mice injected with pLNCX2-B16, the mice inoculated with CCR7-B16 cells developed ear tumors within three weeks, which were accompanied by cervical LN metastasis in 90% of mice at Day 20; (**B**). Kinetics of B16 tumor growth in ears showed that CCR7 expression enhanced tumorigenesis in primary sites in addition to facilitating LN metastasis. (Inoculation number: 100,000 cells/ear) [48].

When limited numbers of tumor cells were inoculated into murine skin, overexpression of CCR7 directly facilitated primary tumor formation (Figure 1) [48]. In the studies by Fang *et al.*, immunological rejection of non-CCR7-expressing B16 melanoma cells was apparent within the first week of tumor implantation, suggesting the CCR7 expression can help tumor cells evade host antitumor responses. Expression levels of interferon-regulated chemokines such as CXCL10 (as well as numbers of CD4+ and CD8+ T cells) were 5-10 fold lower in the CCR7-B16 tumors compared to the control tumors, suggesting that expression of CCR7 was able to dampen the anti-tumor immune responses within the tumor microenvironment. Careful serial comparative analysis of numbers of metastatic cells in the draining LN of mice injected with either CCR7- or control B16 cells showed that significant differences in numbers of metastatic B16 cells did not occur until 14 days or more after inoculation when CCR7-B16 tumors were grossly visible [48]. These data suggested that CCR7 does not immediately enhance LN migration of CCR7-bearing cells. Alternatively, it is possible that the small numbers of tumor cells that do migrate to draining LN in both cases are localized within different regions of the LN, possibly affecting anti-tumor response.

Collectively, these results suggest that CCR7 expression by tumor cells may alter the local immune environment in addition to simply facilitating migration of tumor cells toward lymphatic vessels and LN. Of note, CCR7 has been found to be expressed by many epithelial cancers, including those of the breast, stomach, and colon, suggesting that its role in increasing LN metastasis may be generalizable to other cancers. Its expression has prognostic significance, especially in gastric cancer where it is correlated with LN metastasis [49] and poor patient outcome [50]. Novel engineered protein antagonists of CCR7 that bind CCL21 have been shown to block CCR7-mediated migration of melanoma cells *in vitro* and *in vivo*, raising the potential for therapeutically active CCR7 antagonists in patient care [51].

**Figure 2.**
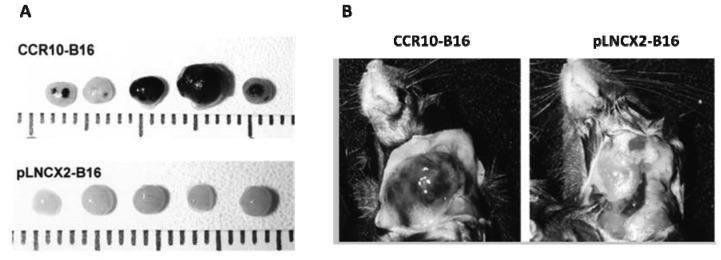
Growth and regional LN metastasis of CCR10- and pLNCX2-B16 tumor cells. (**A**). CCR10- or pLNCX2-B16 cells were injected into the footpads of mice. After 21 d, draining popliteal LNs showed significant degrees of tumor metastasis in CCR10-B16 cells injected mice compared to control mice; (**B**). CCR10- or pLNCX2-B16 cells were injected into the dermis of ear skin in mice. 20 d after inoculation, dissection of the cervical region ipsilateral to the tumor injection sites revealed a large cervical LN metastasis in a CCR10-B16-injected mouse, but not in a pLNCX2-B16-injected mouse Scale bar is calibrated in mm [52].

We have also found that CCR10 can play a role in enhancement of LN metastasis. CCR10 was observed in the majority of cutaneous melanoma primary tumors by immunohistochemistry [52]. Its ligand, CCL27, is constitutively produced in the basal layer of the epidermis but is released under inflammatory conditions [53]. B16 cells that overexpressed CCR10 formed significantly larger primary tumors and more LN metastases than CCR10-negative control cells (Figure 2) [52]. *In vitro*, exposure of CCR10-B16 cells to CCL27 led to rapid activation of Akt, which resulted in resistance of CCR10-B16 cells to cell death induced by melanoma antigen-specific cytotoxic T cells. Hence, CCR10 engagement by locally produced CCL27 facilitates melanoma progression, most likely through activation of PI3K/Akt, which may allow melanoma cells to escape host immune antitumor killing mechanisms [52].

CXCR4 expression has been shown to be an independent clinical prognostic marker in primary cutaneous malignant melanomas [54]. Analysis of CXCR4 expression in melanoma-infiltrated LNs indicated that high CXCR4 expression in LN metastases was correlated with shorter DFS and could be used as a prognostic marker in order to stratify melanoma patients at higher risk of progression [55]. Studies indicated that chemoresistant CD133+ melanoma cells were highly enriched with CXCR4 after chemotherapy thereby enhancing metastatic potential *in vivo* [56]. Interestingly, lymphatic endothelial cells promoted the migratory activity of CXCR4^+^/CD133^+^ tumor cells, but not those of CXCR4 tumor cells. The lymphatic vessels in metastatic tissues stimulated CXCR4^+^/CD133^+^ cell metastasis through the production and release of CXCL12. These results emphasize the shared roles of CXCR4-CXCL12 axis and lymphatic microenvironment in facilitating metastasis of melanoma cells [56]. Early studies have suggested a role for CXCR4 in experimental LN metastasis in a murine breast cancer model [42], but not in a melanoma metastasis model in mice [45].

Of note, malignant melanoma cells also express CCL21, which has been recently revealed to convey a significant, alternative mechanism for tumor progression. CCL21 expression by melanoma tumors in mice has been shown to be associated with an immunotolerant microenvironment, which included the induction of lymphoid-like reticular stromal networks, an altered cytokine milieu, and the recruitment of regulatory T cell populations [57]. CCL21-mediated immune tolerance was dependent on host expression of CCR7 and could protect distant, co-implanted CCL21-deficient tumors and nonsyngeneic allografts from rejection. It is proposed that by altering the tumor microenvironment, CCL21-secreting tumors shift the host immune response from immunogenic to tolerogenic, which facilitates tumor progression [57].

Other chemokines are likely to participate in melanoma progression. CXCL8, acting as an autocrine/paracrine growth factor, influences the process of melanoma progression by activating its receptors, CXCR1 and CXCR2. The expression of CXCL8 in melanoma, as well as its receptors CXCR1 and CXCR2, has demonstrated a positive correlation with disease progression [58]. Over-expression of CXCR1 and CXCR2 in melanoma cells conferred an aggressive phenotype to melanoma cells based on enhanced proliferation, migration, and tumor growth in mice [58,59]. In UVB-irradiated nude mice, CXCL8 was identified as a molecular basis for enhanced tumorigenicity and metastatic potential [60]. CXCL8 and CXCR2 were detected via immunostaining in metastatic lesions (e.g., draining LNs) in human malignant melanoma specimens, supporting their contributions to aggressive growth and metastasis [61].

Beyond the fundamental roles of chemokines in recruiting leukocytes to the tumor microenvironment, studies have shown that chemokine signals contribute to monocyte differentiation. Microarray analysis of primary monocytes revealed that the endothelial growth factor, VEGF, and the angiogenic chemokine, CCL1, were up-regulated in response to CXCL12. Furthermore, primary blood monocytes themselves were shown to secrete CXCL12 and express CXCR4 and CXCR7, which resulted in an autocrine/paracrine loop that modulated differentiation of monocytes towards a distinct program with proangiogenic and immunosuppressive functions [62].

## 7. Lymphangiogenesis Is Highly Correlated with Lymph Node Metastasis

The presence of CCR7 on tumor cells and the accumulated evidence suggesting their involvement in LN metastasis is an appropriate segue way to a discussion of the role of tumor-induced lymphangiogenesis in LN metastasis. After all, it is clear that tumor cells must find and enter lymphatic vessels prior to eventual accumulation in the draining LN. Indeed, clinical evidence suggests that the extent of lymphangiogenesis in primary cutaneous melanoma can be used as a novel prognostic indicator for the presence of sentinel lymph node metastases at the time of surgery [63,64]. In a retrospective study, increased levels of melanoma-associated lymphangiogenesis were found to be inversely correlated with disease-free survival and with overall survival of melanoma patients [65].

Lymphangiogenesis is fairly well understood at the molecular developmental level. The powerful lymphatic-specific growth factors discovered were vascular endothelial growth factor (VEGF)-C and VEGF-D which are known to bind to VEGF-receptor-3 (VEGF-R3) expressed on lymphatic endothelium [66–69]. Since VEGF-C and -D were the first lymphangiogenesis-factors identified, several studies have investigated their possible role in melanoma metastasis and lymphangiogenesis. VEGF-C expression levels were found to be significantly correlated with lymphatic vessel density in primary melanomas [63] and with lymph node metastasis [70]. Significant differences in VEGF-C expression were also observed between nodal metastases and primary melanomas, and between metastatic vs. non-metastatic LN [71]. However, no correlation was found between the expression levels of VEGF-D and the incidence of lymph node metastasis [63,70]. Moreover, VEGF-C overexpressing tumors, including melanoma cell line B16-F10, exhibited an increased incidence of lymphatic metastasis in the cervical LN when implanted in the ear [72]. Therefore VEGF-C appears to represent the most important known lymphangiogenic factor in human cancers, including malignant melanoma.

Very recently, integrin α4β1 signaling has been reported to be required for lymphangiogenesis and tumor metastasis [73]. Fibronectin-binding integrin α4β1 and its ligand fibronectin are novel functional markers of proliferating lymphatic endothelium. Genetic loss of integrin α4β1 expression in mice blocks growth factor and tumor-induced lymphangiogenesis as well as tumor metastasis to lymph nodes [73]. Rebhun *et al.* reported that α4 integrin expressing B16-F1 tumors metastasized to lymph nodes ∼2.5-times more frequently than B16-F1 tumors that expressed low levels of this integrin, suggesting a role for this adhesion pathway in melanoma metastasis [74].

## 8. Inflammation-Promoted Epithelial to Mesenchymal Transition (EMT) in LN Metastasis

Recent studies focusing on the molecular pathways that underlie the role of inflammation and cancer point to the epithelial to mesenchymal transition (EMT) as a common link in cancer progression. The occurrence of EMT during tumor progression allowed tumor cells to infiltrate surrounding tissue and to ultimately metastasize to distant sites [75]. There are many molecules that are associated with EMT, and some of the players can be used as biomarkers for this transition. Alonso *et al.* compared the gene expression profiling between metastatic and nonmetastatic melanoma cases and identified 243genes (206 up-regulated and 37 down-regulated) as biomarkers for EMT. Using tissue microarrays, the authors confirmed that the expression of a set of proteins included in the EMT associated gene group (N-cadherin, osteopontin, and SPARC /osteonectin) was significantly associated with metastasis development [76]. Defining features of EMT include a reduction in E-cadherin levels, which was shown in melanoma [77] and a concomitant increase in N-cadherin expression [75]. Other factors included the Snail1 transcriptional repressor, a member of the zinc-finger protein family, which is known to play critical roles cell survival, adhesion, and migration [78].

Regulatory cytokines such as TNF-α and TGF-β also play important roles in EMT. TNF-α is crucial for the induction of NF-κB, the major inflammatory response pathway [79] that directly activates expression of potent EMT inducers, including Snail1 and Zeb [80–85]. TGF-β1 is also produced by cancer cells as well as by stromal cells in what may be a regulatory response to control inflammation in the tumor microenvironment [86]. TGF-β1, however, induces EMT and thus facilitates invasion and dissemination [87]. In melanoma, it is known that melanoma cells express TGF-β1, -β2, and -β3b [88]; TGF-β1 and TGF-β2 plasma levels are increased at later stages of melanoma development [89,90]. This finding suggests that these inflammatory cytokines can also take part in EMT in melanoma cells.

## 9. Summary

**Figure 3.**
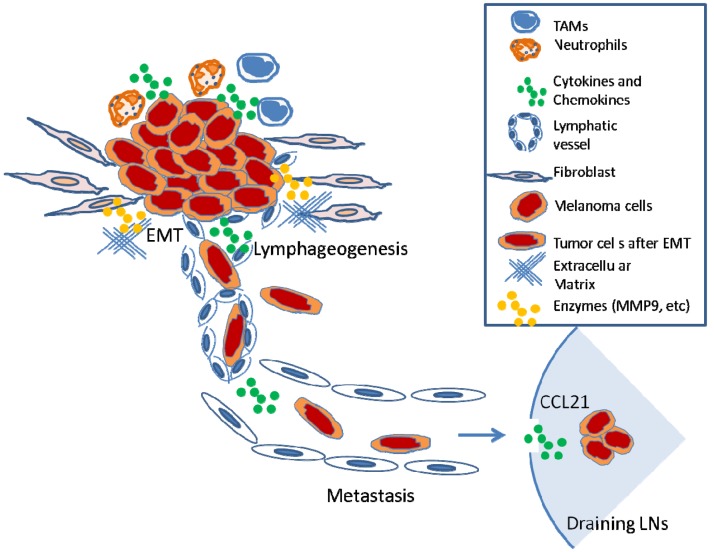
Model of melanoma cell metastasis to regional LNs. The process of inflammation-mediated metastasis of melanoma cells from primary sites to regional LNs. (1). Tumor cells induce an inflammatory microenvironment (*i.e.*, infiltration of tumor-associated macrophages (TAMs) and neutrophils; production of cytokines and chemokines); (2). Facilitated by matrix-metallo proteases, tumor cells invade basement membrane and degrade extracellular matrix; (3). Tumor cells undergo epithelial to mesenchymal transition (EMT), which increases the propensity of metastasis; (4). Increased numbers of lymphatic vessels around or with tumors increase probability of invading lymphatic system; (5). Tumor cells passively flow within lymphatic vessels into draining LNs; (6). Metastatic cells survive and form focal metastases within LN.

In summary, diverse biological processes, including inflammation, EMT, and lymphangiogenesis, influence nodal metastasis. Pre-existing or tumor-elicited inflammation assists the tumor metastasis at almost every step necessary for LN metastasis (*i.e.*, malignant cell expansion, invasion, EMT, migration, and microenvironment modulation) (Figure 3). Many of the newly identified genes that appear to influence LN metastasis facilitate general motility or invasion properties that promote growth of tumors, encourage transition to a more motile phenotype, or increase the ability of a cancer cell to survive at distant metastatic sites. Already, the identification of such biomarkers allows physicians to better predict patient survival. Investigators are beginning to understand that certain receptors such as CCR7 not only promote LN metastasis but may also profoundly affect tumorigenesis. The development of CCR7 antagonists will allow investigators to determine whether such agents can potentially be beneficial to cancer therapy.
